# Home-based pulmonary rehabilitation in patients undergoing (chemo)radiation therapy for unresectable lung cancer: a prospective explorative study

**DOI:** 10.1007/s11547-022-01562-w

**Published:** 2022-10-10

**Authors:** Paolo Borghetti, Jacopo Branz, Giulia Volpi, Simone Pancera, Riccardo Buraschi, Luca Nicola Cesare Bianchi, Marco Lorenzo Bonù, Diana Greco, Giorgio Facheris, Cesare Tomasi, Laura Pini, Michela Bezzi, Salvatore Grisanti, Maria Sole Gallazzi, Andrea Borghesi, Michela Buglione di Monale e Bastia, on behalf of the ASST Spedali Civili of Brescia Lung Unit

**Affiliations:** 1 Radiation Oncology Department, ASST Spedali Civili and University of Brescia, P.le Spedali Civili, 1, 24123 Brescia, Lombardia Italy; 2 IRCSS Fondazione Don Carlo Gnocchi, Milan, Italy; 3 Respiratory Medicine Unit, Deparment of Clinical and Experimental Sciences, ASST Spedali Civili, University of Brescia, Brescia, Italy; 4 Division of Pneumology, University Hospital ASST Spedali Civili of Brescia, Brescia, Italy; 5 Medical Oncology Unit, ASST Spedali Civili and University of Brescia, Brescia, Italy; 6 Thoracic Surgery Unit, Cardiothoracic Department, Spedali Civili, Brescia, Italy; 7 Department of Medical and Surgical Specialties, Radiological Sciences and Public Health, University of Brescia - ASST Spedali Civili of Brescia, Brescia, Italy

**Keywords:** Lung cancer, Stage III NSCLC, Radio-chemotherapy, Exercise training, Pulmonary rehabilitation, Home-based rehabilitation program

## Abstract

**Aims:**

The prevention of pulmonary toxicity is an important goal for patient candidate to radiation therapy for lung cancer. There is a lack of evidence on the role of exercise training for patients with unresectable stage III lung cancer candidated to radical treatment. The aim of this study was to evaluate the feasibility of a home-based pulmonary rehabilitation (PR) program and to identify reliable tools in terms of respiratory function, exercise capacity and quality of life.

**Methods:**

Patients’ recruitment lasted from April 2020 till February 2022. The PR program was proposed concomitantly to radiation therapy to the first 20 patients (interventional group, IG), and the other 20 patients were identified as an observational group (OG). All patients were assessed at baseline (T0) and after 8 weeks (T2) with 6 minute walking test (6MWT), modified Borg Scale (mBORG), SF-36 questionnaire (SF-36) and pulmonary function test (PFT); after 4 weeks (T1), only SF-36 was administered.

**Results:**

A decrease of 13.8 m in the walked-distance was registered in the OG between T0 and T2 (*p* = 0.083). Instead, an increase of 56.6 m in the distance walked was recorded in the IG between T0 and T2 (*p* ≤ 0.001).

In the OG, the mBORG scores showed a negative trend. On the contrary, in the IG, these scores showed a slight improvement. In the OG, all the items of SF-36 scores decreased between T0 and T1. In the IG, an increased trend from T0 to T2 was observed for all the items of SF-36. No clinically significant variations were detected from baseline to T2 in both groups regarding PFT.

**Conclusion:**

The 6MWT, mBORG and SF-36 resulted as useful tools to assess the role of a PR program. A significant gain in functional exercise capacity and a prevention of the physiological impairment of QoL during radio(chemo)therapy was registered.

## Introduction

Lung cancer is the second most common cancer in both men and women, and it is by far the leading cause of cancer death, making up almost 25% of all cancer deaths. [1].

Non-small cell lung cancer (NSCLC) accounts for approximately 85% of all histologies [2] and approximately 30% is considered locally advanced disease at diagnosis [3] and collocated in stage III (AJCC Cancer Staging Manual, Eighth Edition). Small cell lung cancer (SCLC) comprises 15% of lung cancers and 25% to 30% of these have limited disease (LD) at diagnosis [4]. In patients with stage III unresectable NSCLC, the standard of care is concurrent radio-chemotherapy (CCRT) plus consolidation with Durvalumab for 1 year [3]. Also, for patients with limited disease, SCLC, the standard therapy is based on four cycles of chemotherapy concurrent with thoracic radiotherapy [5].

Data from meta-analysis show that severe esophagitis is the only side effect with a significantly higher rate in CCRT as compared to sequential chemo-radiotherapy (SCRT) [6], despite improvements in radiotherapy techniques and supportive care have reduced the occurrence of this complication [7]. However, in the clinical practice, CCRT is often considered less tolerated, and other side effects, such as respiratory disorders, can potentially negatively affect the patient's quality of life (QoL) and general health [8]. The Pacific trial reported an incidence of all grade pneumonitis and pneumonia of 34% and 13%, respectively, for the group treated with durvalumab as maintenance, as compared to 25% and 8% in the placebo arm [3]. Therefore, a careful patient’s selection and risk assessment is essential prior to candidate patients to a radical treatment option [8].

In this scenario, an emerging interest in supportive care during active treatment is growing up [9]. Historically, patients with cancer were advised to rest and save energy but starting in the late 1980s, new evidence emerged to support a benefit related to physical activity [11].

Physical activity can play a beneficial role during and after oncological treatments, getting improvements in physical fitness [11, 12] (aerobic, strength, flexibility), health-related quality of life (HRQoL) [13], treatment-related side effects [14] and psychological outcomes. [11].

Several studies have already shown that exercise training improved exercise capacity and QoL in patients with lung cancer who underwent surgery [15]. Exercise studies in the pre- and post-operative setting showed an improvement in physical performance and cardiorespiratory fitness. [16, 17, 18, 19, 20]. In contrast, there is a lack of evidence of the effect of exercise training in unresectable patients candidate to (chemo)radiation therapy.

The aim of this study was to evaluate the feasibility of a home-based pulmonary rehabilitation (PR) program in patients with stage III unresectable NSCLC or LD-SCLC treated with radical chemo-radiotherapy and to identify reliable tools able to test the effectiveness of the program in terms of respiratory function, exercise capacity and HRQoL.

## Material and methods

### Trial design

The trial was designed as prospective and exploratory since the absence of available data concerning the efficacy of a PR program for unresectable stage III NSCLC or LD-SCLC.

### Participants

To be eligible, participants were to be older than 18 years, male or female, with histological diagnosis of NSCLC or SCLC, subject to radiation therapy with curative intent (60 Gy, 2 Gy per fraction, 5 daily fractions per week, in both NSCLC and SCLC). All the patients were treated with volumetric modulated arc therapy (VMAT) and image-guided radiation therapy (IGRT) techniques. Also, eventual chemotherapy was allowed with a regimen platinum-based doublet.

Exclusion criteria were the presence of metastatic disease, severe cardiovascular comorbidities, orthopedic or neurological disorders either limiting physical exercise or understanding of instructions, refusal to informed consent.

### Outcomes

Considering the explorative intent of the study, the outcomes aimed to detect the reliability of the tools applied to test the efficacy of the PR program. Three tests were identified and investigated: 6 min walking test (6MWT) and modified BORG scale, Medical Outcomes Study Short Form (SF-36) Questionnaire and pulmonary function testing (PFT).

#### Six minutes walking test (6MWT)

The functional exercise capacity was assessed as the changes in the distance walked during the 6MWT at 8 weeks as compared to baseline. 6MWT is a practical, simple and cost-effective test. It requires a 30 m indoor walking corridor, with hard and flat floor. Subjects are asked to walk, back and forth in the corridor between two cones, for 6 min at self-paced speed in order to walk as far as possible (without run or jog). The total distance walked (6 Minute Walking Distance – 6MWD) is recorded. In this study, 6MWT was performed according to ATS (American Thoracic Society) guidelines [21]. Dyspnea and perceived exertion were recorded before and after test execution using modified BORG scale (mBORG scale).

#### Medical outcomes study short form (SF-36) Questionnaire

It is a generic patient-reported outcome measure that quantifies health status and measures health-related quality of life. It is composed by 36 items measure divided into eight subscales (Physical Functioning, Role Limitation due to Physical Problem, General Health Perceptions, Vitality, Social Functioning, Role Limitation due to Emotional Problems, General Mental Health and Health Transition). Respondents are asked to answer items referring to past 4 weeks, the 8 summed scores obtained are linearly transformed onto a scale to 0 (negative health) to 100 (positive health) to provide a score for each subscale which can be used independently.

#### Pulmonary function test (PFT)

PFT performed measured forced vital capacity (FVC), maximal expiratory volume in the first second (FEV_1_), and maximal forced expiratory flows and different lung volumes were measured using a bell spirometer in sitting position (COSMED Instruments, Italy). Lung diffusion capacity for carbon monoxide (DLCO) and its main determinants, alveolar volume and transfer rate for CO (KCO), were measured by single breath technique (COSMED Instruments, Italy). DLCO and KCO were adjusted for hemoglobin.

The functional parameters considered for the analysis were exclusively FEV_1_ as absolute value and percentage of predicted (pred), FEV_1_/FVC % ratio and DLCO as absolute value.

### Procedure

**Fig. 1:**
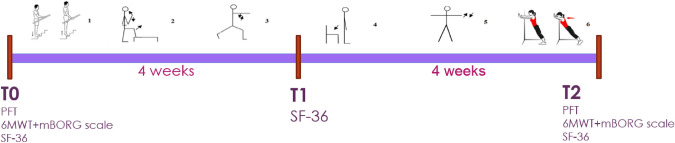
Time to assessment: at b*aseline* calculated 1 week before start radiotherapy (*T0*)*,* after 4 weeks (T1) and at a 1 week after the end of radiotherapy (T2)

Patients’ recruitment started in April 2020 till February 2022 at Radiation Oncology department of the XXX. An arbitrary sample size of 40 patients was calculated to reach the study explorative endpoint. All patients were assessed in different time-points: b*aseline* defined as 1 week before starting radiotherapy (*T0) with* 6MWT, mBorg Scale, SF-36 and PFT; after 4 weeks (T1) only with SF-36 and 1 week after the end of radiotherapy with 6MWT, mBorg Scale, SF-36, PFT (T2) (Fig. 1).

The PR program was proposed to the first 20 patients enrolled (interventional group, IG); the next 20 patients were identified as an observational group (OG).

### Home-based PR program

The 8-week training program included three endurance sessions and two resistance sessions per week, once a day [22, 23, 24] from T0 to T2.

During the first supervised sessions, participants were instructed how to carry out exercises at home by an experienced physiotherapist. Also, participants received a daily diary for recording perceived exertion and dyspnea values before and after each training session. Planned weekly contacts with the physiotherapist took place at patient's home, in hospital after the daily radiotherapy session, or by phone. Other contacts were possible on demand by the patient.

#### Endurance training program

The endurance training consisted of a quick stride walk preceded by 5 min warm-up of walking at normal pace. For the quick stride definition, subjects were instructed to walk fast without resulting in running and try to maintain a score from 3 to 5 of mBorg scale of perceived exertion and dyspnea (moderate-strong). The mBorg Scale is a valuable tool to self-measure perceived exertion and dyspnea during effort [25]. The Modified version is a 11 points scale, from 0 = nothing at all to 10 = very very hard. Subjects were instructed about scale rating system before effort that is graded using numbers and words.

Each week subjects increased their walking time by 5 min, starting from 20 min in the first week, trying to reaching up to 50 min during the eighth week. At the end of each session, subjects performed 10 min session of stretching according to instruction provided: Addressed muscles were triceps surae, hamstrings, quadriceps, pectoralis, scapulae adductors, deltoids and triceps brachii.

#### Resistance training program

**Fig. 2:**
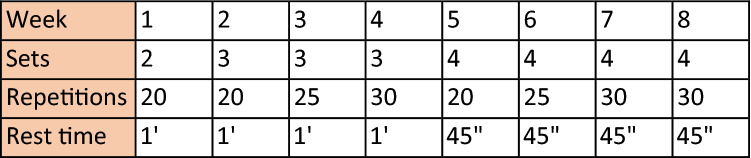
Resistance training progression

Resistance training program was composed of six exercises: calf raise, sit to stand, up-down stepper, arm abduction/adduction, lunges, wall push-ups. Subjects were instructed to correctly perform the exercises by the physiotherapist. Training load was increased as shown in Fig. 2. One repetition is a single exercise; a series of repetitions, performed consecutively one after the other, constitutes a set. Over the course of the 8 weeks, the repetitions of exercises gradually increase; as well as the number of series to perform. The recovery time gradually decreases. Each session was preceded by 2 min warm-up exercise (1 min of crunches, 1 min of bridge exercise) and followed by 10 min of stretching, as described previously for endurance training.

### Statistical analysis

According to the explorative nature of the study, a descriptive analysis to quantify possible variation in 6MWT, mBORG Scale, SF-36 and the PFT at T0, T1 and T2 either for patients undergoing to PR program or not was elaborated. The Kolmogorov–Smirnov test was used for the continuous variables to define if distribution resulted normal or not and consequently to apply *t* test or Wilcoxon test, respectively. The chi-square test was adopted for categorical variable. A level of significance set at *p* value < 0.05 was used for comparison between groups. The statistical analysis was performed using IBM SPSS™ software version 25.0.2 (IBM SPSS Inc. Chicago, Illinois) and Stata™ software release 15.0 (Stata Corporation, College Station, Texas).

## Results

### Study population

**Fig. 3:**
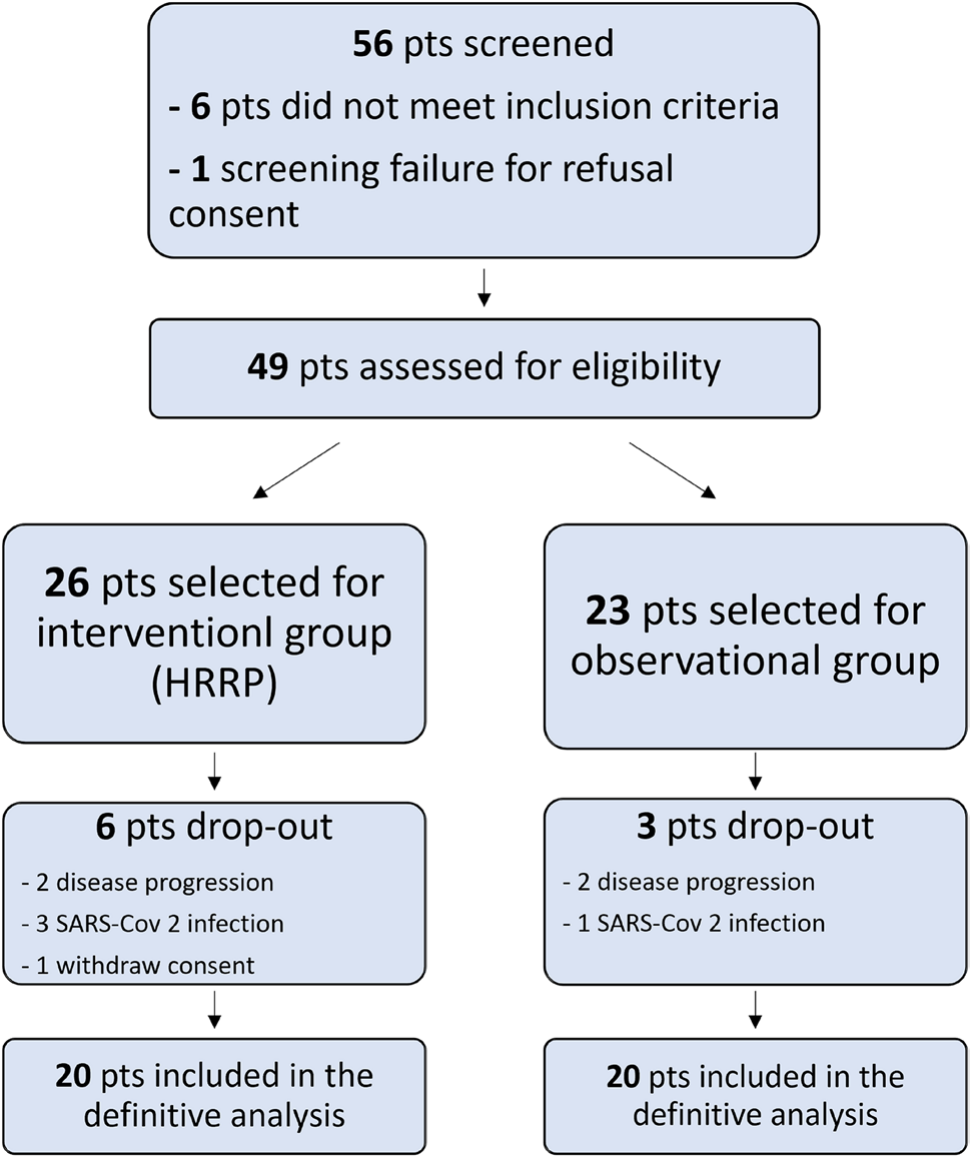
Participant flow through the trial

During the accrual period, 56 patients were screened. One patient was excluded, as screening failure, due to the refusal of informed consent and six patients did not meet the inclusion criteria. Nine patients were considered drop out: one for withdrawing the informed consent, four for SARS-Cov 2 infection and four for disease progression during CCRT (Fig. 3).

Patients’ clinical and therapeutic features are summarized in Table 1. The median age was 68 years (range 50–82), and 21 patients (52.5%) were male. Fifty percent of patients in the IG and 45% of patients in the OG presented COPD.

**Table 1:** clinical and therapeutic features of the patients

	Interventional group *N* (%)	Observational group *N* (%)	Total	*p*
Age				0,190
≤ 65 y	10 (50%)	6 (30%)	16 (40%)	
> 65 y	10 (50%)	14 (70%)	24 (60%)	
Sex				0,342
Male	9 (45%)	12 (60%)	21 (53%)	
Female	11 (55%)	8 (40%)	19 (47%)	
Education				0,321
Primary school	3 (15%)	8 (40%)	11 (27%)	
Secondary school	11 (55%)	7 (35%)	18 (45%)	
High school	4 (20%)	4 (20%)	8 (20%)	
Graduation	2 (10%)	1 (5%)	3 (8%)	
Smoking status				0,413
Never	0	1 (5%)	1 (3%)	
Current	9 (45%)	6 (30%)	15 (37%)	
Former	11 (55%)	13 (65%)	24 (60%)	
COPD				0,630
No	10 (50%)	11 (55%)	21 (52.5%)	
Grade 1	4 (20%)	6 (30%)	10 (25%)	
Grade 2	5 (25%)	2 (10%)	7 (17.5%)	
Grade 3	1 (5%)	1 (5%)	2 (5%)	
Comorbidity Index				0,482
≤ 4	7 (35%)	6 (30%)	13 (32.5%)	
> 4	13 (65%)	14 (70%)	27 (67.5%)	
Histology				0,480
NSCLC: squamous cell carcinoma	6 (30%)	7 (35%)	13 (32%)	
NSCLC: adenocarcinoma	13 (65%)	10 (50%)	23 (58%)	
SCLC	1 (5%)	3 (15%)	4 (10%)	
Stage (sec. WHO 8° ed.)				0,849
IIB	2 (10%)	1 (5%)	3 (8%)	
IIIA	5 (25%)	6 (30%)	11 (27%)	
IIIB	12 (60%)	11 (55%)	23 (57%)	
IIIC	1 (5%)	2 (10%)	3 (8%)	
Treatment				0,244
RTT alone	2 (10%)	0	2 (5%)	
Sequential RCT	3 (15%)	3 (15%)	6 (15%)	
Concurrent RCT	15 (75%)	17 (85%)	32 (80%)	
	Mean (SD)	Mean (SD)	Mean (SD)	
PTV (cm3)	435.5 (255.3)	367.43 (113.4)	401 (198.0)	0,283
Lung V5 (%)	52.3 (8.1)	51.3 (6.1)	51.8 (7.1)	0,677
Lung V20 (%)	20.6 (3.8)	23.2 (3.8)	21.9 (4)	0,420
Lung mean (Gy)	14.8 (2.7)	15.2 (2.2)	15.0 (2.4)	0,671

COPD, chronic obstructive pulmonary disease; RT, radiotherapy; SCRT, sequential chemo-radiotherapy; CCRT, concurrent chemo-radiotherapy; PTV, planning target volume

Thirty percent of patients in the IG had COPD staged as Global Initiative for Chronic Obstructive Lung Disease (GOLD) stage 3 or 4, compared to 15% of patients in the OG. Thirteen patients (65%) in the IG and 14 (70%) in the OG had a Charlson Comorbidity Index greater than 4. Nineteen patients (95%) in the IG and 17 (85%) in the OG had NSCLC; only one patient (5%) in the IG and three (15%) in the OG had SCLC. Most of the patients in both groups underwent CCRT (75% and 85% in the IG and the OG respectively); two patients (10%) in the IG and none in the OG received exclusive radiotherapy. All the patients received volumetric modulated arc radiotherapy (VMAT) and image-guided radiation therapy. The dose constraints to lungs and heart were respected according to Institutional policy. No statistically significant differences were detected between groups regarding age, sex, education, smoking status, COPD, Charlson Comorbidity Index, histology, stage disease, treatment, and dosimetric parameters at baseline (Table 1).

6MWD, mBORG scale and PFT results for OG and IG are summarized in Table 2.

**Table 2:** 6MWD, mBORG scale and PFT results for OG and IG

	Observational group											Interventional group										
	T0					T2					*p*	T0					T2					*p*
	Mean	Median	SD	Min	Max	Mean	Median	SD	Min	Max		Mean	Median	SD	Min	Max	Mean	Median	SD	Min	Max	
6MWD	434,4	434,5	65,3	285	545	420,6	437,5	74	250	540	0,083	411,6	440	122,9	110	568	468,2	497,5	93,1	295	600	0,000
D pre	0,45	0	0,8	0	3	0,6	0	1,1	0	4	0,623	0,95	0	1,5	0	4	0,7	0	1,3	0	5	0,509
F pre	0,2	0	0,7	0	3	0,3	0	0,9	0	4	0,854	0,7	0	1,3	0	5	0,4	0	0,9	0	3	0,129
D post	2,8	2	1,4	1	6	3,8	3,5	1,8	1	8	0,02	3,9	3,5	1,8	2	8	3	3	1,3	1	6	0,08
F post	2,1	2	1,6	0	5	2,7	3	1,8	0	6	0,074	3,1	2.5	2,6	0	10	2,4	2	1,7	0	6	0,218
FEV1 (l)	2,2	2.3	0,6	1,2	2,9	2,3	2,4	0,7	1,2	3,3	0,107	2,0	1,8	0,8	0,7	3,7	2,1	2	0,7	1,3	4,0	0,337
FEV1 (%)	93,6	91	18,9	43	118	96,6	97	18,2	42	125	0,128	85	83	27,8	36	158	91	88	25,5	39	147	0,158
FEV1/FVC	67,6	71,5	12,8	28	82	69,7	73	12,3	29	79	0,008	66,4	67	11,4	4	89	65,6	_66_	10,8	49	81	0,810
DLCO (ml/mmHg/min)	15,6	15,3	4,7	6,9	26,4	13,6	12,7	4	8,3	22,4	0,008	15,5	15	5,4	6,9	29	15,3	15	4,3	6,1	23,7	0,654

#### Minutes Walking Test (6MWT) and Modified BORG Scale for dyspnea and exertion perceived

A decrease of 13.8 m in the distance walked was registered in the OG between T0 and T2 (434.4 m vs. 420.6, respectively) (*p* = 0.083). Instead, an increase of 56.6 m in the distance walked was recorded in the IG between T0 and T2 (411.6 m vs. 468.2 m) (*p* = 0,000).

The scores recorded at modified BORG scale showed a negative trend, for all the parameters, in the OG: from 0.45 at T0 to 0.60 at T2 for dyspnea pre-6MWT (p = 0,623); from 0.20 at T0 to 0.30 at T2 in fatigue pre-6MWT (*p* = 0.854); from 2.8 at T0 to 3.8 at T2 in dyspnea post-6MWT (*p* = 0.02); from 2.1 at T0 to 2.7 at T2 in fatigue post-6MWT (*p* = 0.074). On the contrary, in the IG, these scores showed a slight improvement: from 0.95 at T0 to 0.70 at T2 for dyspnea pre-6MWT (*p* = 0,509); from 0.7 at T0 to 0.40 at T2 in fatigue pre-6MWT (*p* = 0.129); from 3.9 at T0 to 3.0 at T2 in dyspnea post-6MWT (*p* = 0.08); from 3.10 at T0 to 2.4 at T2 in fatigue post-6MWT (*p* = 0.218).

### Medical Outcomes Study Short Form (SF-36) Questionnaire

In the OG, decreased scores were recorded between T0 and T1 regarding all the items: physical functioning (from 78 to 64), role limitation due to physical health (from 46 to 33), role limitation due to emotional problems (from 60 to 45), energy/fatigue (from 61 to 52), emotional well-being (from 71 to 65), social functioning (from 84 to 70), pain (from 71 to 70) and general heath (from 57 to 56). This decrease was partially recovered from T1 to T2 (physical functioning reached 73, role limitation due to physical health 34, role limitation due to emotional problems 62, energy/fatigue 55, emotional well-being 70, social functioning 77, pain 70 and general health 58). The declined scores resulted significant for physical functioning either for T0–T1 interval or T1–T2 interval (*p* = 0.005 and *p* = 0.049, respectively); energy/fatigue for T0–T1 (*p* = 0.023) and social functioning for T0–T1 (*p* = 0.024).

The scores registered at T0-T1-T2 in the IG were 79-81-81 for physical functioning; 48-58-61 for role limitation due to physical health; 45–62–57 for role limitation due to emotional problems; 61-58-61 for energy/fatigue; 61-68-68 for emotional well-being; 73-76-80 for social functioning; 78-77-76 for pain and 55-54-60 for general heath. The increase trend observed in every item in the IG is statistically significant only for general health for T1–T2 interval (*p* = 0.006).

**Fig. 4:**
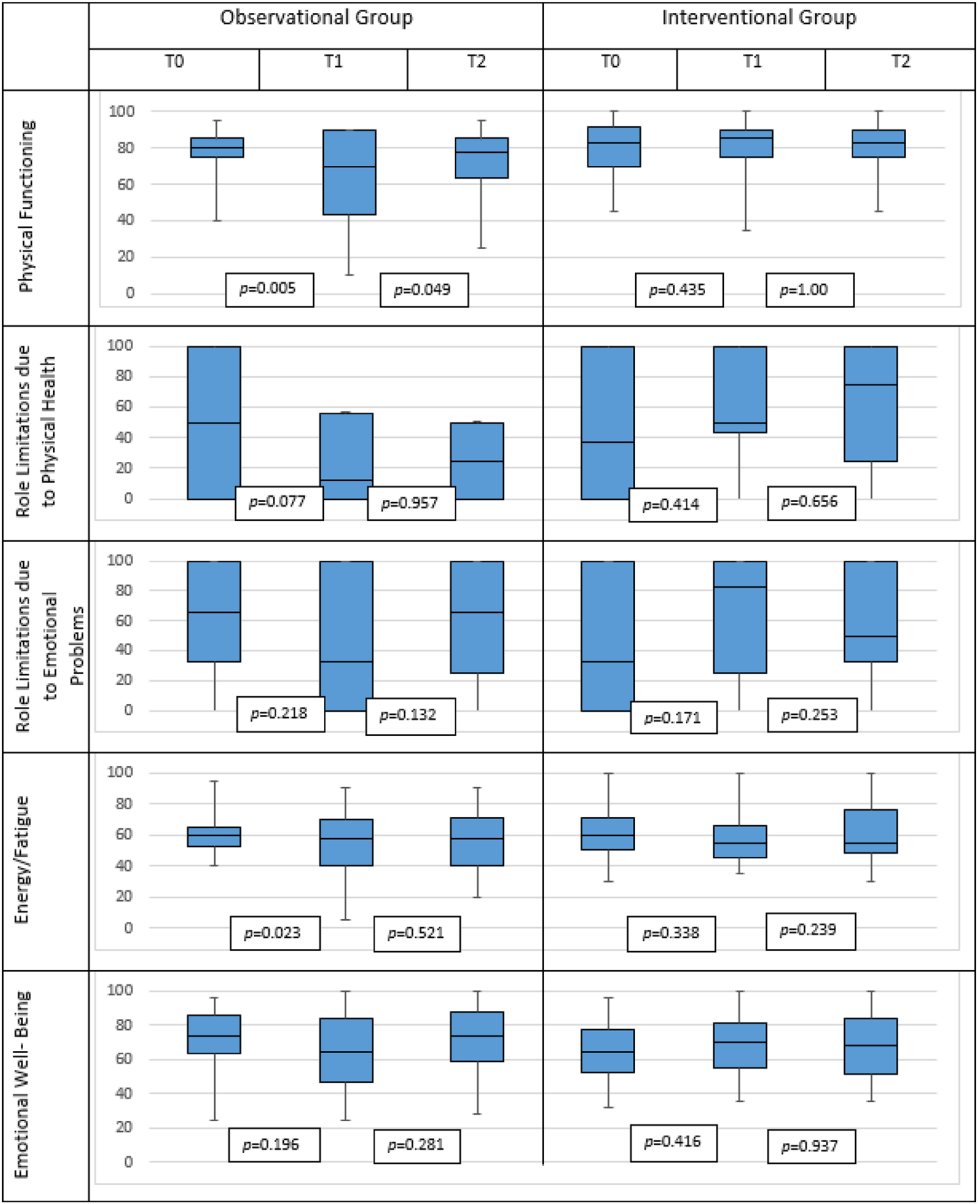

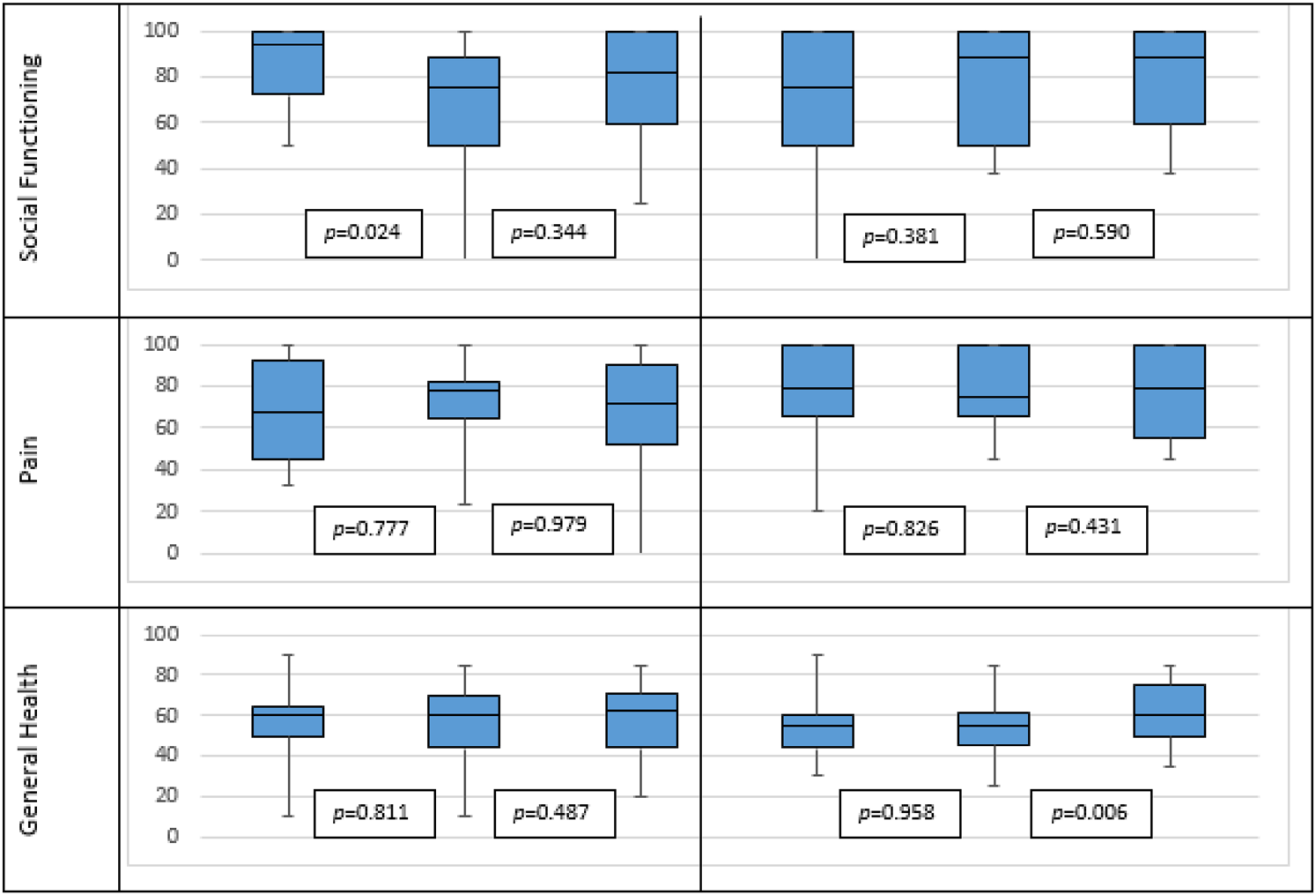
Distribution through T0–T1–T2 of the SF-36 Item in the observational group and the interventional group

All the above-mentioned scores were reported as mean values, Fig. 4 summarizes the results (in the box plots that include median, minimum and maximum values and first and third quartile). All p values reported in Table 2 are referred to nonparametric test for all the items but the energy/fatigue and emotional well-being.

### Pulmonary function test (PFT)

No differences were detected in the FEV_1_ and FEV_1_%pred values from T0 to T2 in both groups.

FEV_1_/FVC % ratio registered was 67.7 at T0 and 69.7 at T2 in the OG (*p* = 0.008), while in the IG was 66,4 at T0 and 65,6 at T2 (*p* = 0.81). The DLCO registered in the OG was 15.6 ml/(min*mmHg) and 13.6 ml/(min*mmHg) at T0 and T2, respectively (*p* = 0,008); in the IG, it resulted 15.5 ml/(min*mmHg) at T0 and 15.3 ml/(min*mmHg) at T2 (*p* = 0,654).

## Discussion

Locally advanced lung cancer is a severe and highly symptomatic disease, presenting symptoms such as dyspnea, weight loss, cough, pain, fatigue, sleep disturbance [26]. At diagnosis, mostly of these patients are old and with a smoking history and therefore often suffering of COPD and cardiovascular comorbidities. Nowadays, the treatments offered to these patients are more and more challenging since the addition of immunotherapy after CCRT.

These integrated treatments are burdened with major toxicities that affects all organ systems and may cause acute and permanent side effects [9]. Supportive care is therefore of utmost importance in this clinical setting. Recently, a group of experts from the European Society for Radiotherapy and Oncology (ESTRO) and the European Society of Medical Oncology (ESMO) identified some items of importance for further improvement of supportive care such as smoking cessation, nutritional supplementation, physical exercise before and during CCRT, prevention and treatment of acute esophagitis, treatment of cough and dyspnea, prophylaxis of nausea, treatment and prevention of cardiac disease and damage. Moreover, an optimization of radiotherapy techniques and chemotherapy adjustments were investigated to reduce toxicity in the era of immunotherapy [9]. In particular, the group of experts concluded that exercise and resistance training improve and restore functional exercise capacity, and it should be offered to patients before and during CCRT [9]. Despite these indications, translation in the clinical practice has not yet been achieved.

Several studies have shown a beneficial impact of preoperative rehabilitation in patients with early-stage NSCLC. A review published in 2020 showed that people with NSCLC who exercised before lung surgery had 67% less risk of developing a postoperative lung complication, a chest drain for fewer days (3 days less), a shorter length of hospital stay (4 days less), better 6‐min walk distance (18 meters more) and lung function before surgery (3% better) [27].

In post-surgical setting, the effects of exercise training on exercise capacity and adverse events are also widely described. Cavalheri et al. published in 2019 a review including eight randomized controlled trials and involving 450 participants. The findings of this review showed an improvement in exercise capacity expressed as the peak rate of oxygen uptake (VO2peak) and in 6MWD in the intervention group [28].

In contrast, studies investigating the effects of physical exercise in patients with advanced lung cancer are rare: In this palliative scenario, a rehabilitation program addressed to metastatic patients showed an advantage in physical fitness and exercise capacity [29], but no benefit on physical functioning HRQoL, dyspnea, fatigue, anxiety and depression [30].

Finally, in the locally advanced lung cancer patients treated with radio-chemotherapy, the evidence is even weaker. A randomized controlled trial tried to explore the impact of a rehabilitation training program but also patients surgically treated were included and the program started after the end of radical treatment [31].

For the reasons described above, the rationale of the study was to investigate the role of a structured home-based PR program consisting of exercise and strength training in the specific setting of patients with locally advanced unresectable lung cancer undergoing radio-chemotherapy.

Since the lack of data, this exploratory study was conducted to identify the tools that could evaluate the effectiveness of the rehabilitation program in terms of respiratory function, exercise capacity and HRQoL in a selected series of patients affected by locally advanced, unresectable, lung cancer candidate to radio-chemotherapy. All the eligible patients completed the PR program but nine, four for disease progression and four for COVID-19. Only one patient withdrew consent. The entire cohort of 20 patients in the IG completed the PR program. The purpose of our study was partially achieved because three of the four tests were found to be effective in detecting an impact on the performance of exercise capacity and HRQoL.

A deterioration in exercise capacity and QoL measured with the 6MWT, the Modified BORG Scale and the SF-36 Questionnaire was observed in the OG group. In the IG, a significant gain in exercise capacity was registered. In the literature, there is not a univocal definition of minimum clinically important distance in 6MWT, however, the result of this study (an increase of 56.6 m) is significant either for the definition suggested by Holland and Granger of 25 m and 42 m, respectively [32]^33^.

All the items of SF-36 questionnaire were increased in the IG, presenting some statistically significant results. There was no decrease in the interim evaluation that is recorded in the OG, leading to the assumption that the home rehabilitation intervention is effective to prevent the physiological impairment of QoL during radio-chemotherapy. These results are consistent with a previous reported data. A study on patients with lung cancer undergone to a home-based walking exercise program showed a positive effect on anxiety and depression [34] and a systematic review including 16 randomized controlled trials with different cancer histology (colorectal, lung, prostate, breast and lymphoma) concluded that physical activity significantly improved QoL during and after medical treatment [35].

Moreover, both the 6MWT, mBORG and SF-36 Questionnaire turned out to be economic tools, easily administered even by non-specialized personnel, easy to understand and to perform.

On the other hand, the PFT was not conclusive maybe due to the short time between the end of the treatment and PFT at T2 time. The FEV1/FVC ratio resulted slightly, but significant, improved in the OG however within the obstruction range. No significant variations were detected from baseline to T2 in both groups in terms of FEV1. The mean value of DLCO significantly decreased in OG, while it remained stable in IG. It is reasonable to assume that the disease has just started to respond, and the lung damage has not yet healed. PFT remains an essential instrument useful to be performed at baseline to assess the patient, but likely it should not be used as a tool for assessing the impact of a home-based PR program. Regarding pulmonary function in a meta-analysis by Salcedo et al. [36] including 21 randomized controlled trials, a small but significant improvement in spirometry value was registered in patients with chronic lung disease such as COPD that underwent to a whole-body exercise training (On average, the training interventions took place over 13.6 ± 12.0 weeks, with 4.0 ± 2.0 sessions per week). In lung cancer setting, the impact of exercise in respiratory function is controversial: In the first year after surgery, patients can experience an increase in pulmonary parameters that may be attributed to compensatory mechanism, such as the expansion of the remaining lobes and vascular tissue [37, 38]. Pulmonary function changes in patients treated with radio-chemotherapy who underwent to an exercise program remain unexplored.

Therefore, this is a very existing topic: Despite the evidence of the feasibility and efficacy of physical exercise in cancer patients, it is urgent to identify personalized rehabilitation programs that meet the needs of this particular category of patients. A phase II randomized controlled trial is ongoing in inoperable NSCLC patients aimed to assess the efficacy of home-based multi-disciplinary exercise and supportive care on change in functional exercise capacity (6MWT), HRQoL measured using Functional Assessment of Cancer Therapy-Lung and the Assessment of Quality of Life [39].

This single-center prospective study managed to complete a rehabilitation program on 40 patients, with a high adherence, considering also the severe impact of COVID-19 pandemic scenario in our center [40]. The compliance at the rehabilitation program is likely due to the feasibility of the intervention itself, the home setting of the training, the availability of a physiotherapist’s support. In addition, the patients were evaluated weekly by the clinician, with the possibility of telephone contact in case of need.

## Conclusion

The exploratory nature of the trial design is responsible of two limitations of this study. The former is the lack of a statistical comparison of the outcomes between the two groups due to the aim of the study and the non-randomized selection of the patients which would make the analysis not methodologically correct. The latter is the impossibility to analyze any correlations between the home-based PR program and the incidence or the management of treatment-related toxicities. Despite this, the encouraging results of this study could constitute a valid basis for future investigations with a more mature trial design. Moreover, with a longer follow-up, it might be possible to assess if the impact of improvement in QoL and exercise capacity will translate in a relevant clinical benefit such as reduction in pulmonary toxicities and improving of tolerability to immunotherapy.
